# Half a Century in Bed

**Published:** 1854-11

**Authors:** 


### HALF A CENTURY IN BED.

Susan Pierson, of Bridgehampton, Long Island, died Feb. 24th, "in her 72d year, *and the* 52*d of her extraordinary confinement.*" Thus was announced, in the Observer of April 27th, the death of one of the "excellent of the earth."

Her case was peculiar ; it is probable it has no parallel. For more than fifty years she did not set her foot upon the floor, and in all that time did not sit upright in bed. One year of that time was spent at a neighbor's house, with which exception the extent of her travels in fifty years was from one corner of her room to another, once a week, in some strong man's arms. This change was always attended with an almost entire loss of voice, from which she did not recover until after a night's repose. The best medical skill and all her patrimony were expended in vain, in endeavors to restore her to health. The upright posture always and immediately produced violent retching. All hopes of her being restored to her former health were long since abandoned.

All who knew the deceased knew her as "*Aunt Susie,*" and all who knew her, knew an humble, truthful, *cheerful* child of God. It was the privilege of the writer as her friend, as it was his duty (a delightful duty,) as her pastor, to see her frequently. Rarely, if ever, has he seen more strongly developed these two traits of Christian character, viz: adoring views of God and humble views of herself.

All her property being consumed, she was dependent. It was touching to hear her speak in gratitude of the goodness of God in providing her so good a home and so many mercies.

For the most part she did not suffer pain. She had all the time of her confinement, excepting one year, the untiring attentions of an inseparable sister, a Christian woman who survives her, about 80 years of age.

"Aunt Susie" lived a quiet, retired life, but not an idle nor a useless life. She was industrious in the use of her knitting-needles, almost her only employment. Her Bible was her constant companion, and was not out of her hands or out of reach for half a century. Three successive pastors found it refreshing and encouraging to retire from the more public duties and cares of their office to spend an hour with her. Many a time has the writer gone from such interviews with her and her sister, more inclined to and better fitted for his labors. She was *useful*,—for she appreciated the word of God,—she stayed up the hands of her pastors,—she rejoiced in the conversion of sinners,—and she was an instance of the divine faithfulness, a monument of God's mercy and a trophy of his grace.

She has gone where there is no sickness.

The New York Observer, from which the above was taken, adds its testimony to its truth, and this history is given in these pages, to encourage any invalid reader of the Journal to labor for "Aunt Susie's" faith and patience, as well as to shame any of them who may be disposed to complain of such little sufferings as may fall to their lot, and which in comparison are scarcely deserving of mention. No doubt all she passed through was necessary to fit her for some elevated sphere in immortality; and now attained, how can she look back upon earth, and all its history connected with her, and count it as not worthy to be named. Reader, rather be thankful that so much less is imposed on you, and even that little, for good.

